# Point-of-Care Multi-Organ Ultrasound Improves Diagnostic Accuracy in Adults Presenting to the Emergency Department with Acute Dyspnea

**DOI:** 10.5811/westjem.2015.11.28525

**Published:** 2016-01-12

**Authors:** Daniel Mantuani, Bradley W. Frazee, Jahan Fahimi, Arun Nagdev

**Affiliations:** * Alameda Health System Highland Campus, Department of Emergency Medicine, Oakland, California; † University of California San Francisco, Department of Emergency Medicine, San Francisco, California

## Abstract

**Introduction:**

Determining the etiology of acute dyspnea in emregency department (ED) patients is often difficult. Point-of-care ultrasound (POCUS) holds promise for improving immediate diagnostic accuracy (after history and physical), thus improving use of focused therapies. We evaluate the impact of a three-part POCUS exam, or “triple scan” (TS) – composed of abbreviated echocardiography, lung ultrasound and inferior vena cava (IVC) collapsibility assessment – on the treating physician’s immediate diagnostic impression.

**Methods:**

A convenience sample of adults presenting to our urban academic ED with acute dyspnea (Emergency Severity Index 1, 2) were prospectively enrolled when investigator sonographers were available. The method for performing components of the TS has been previously described in detail. Treating physicians rated the most likely diagnosis after history and physical but before other studies (except electrocardiogram) returned. An investigator then performed TS and disclosed the results, after which most likely diagnosis was reassessed. Final diagnosis (criterion standard) was based on medical record review by expert emergency medicine faculty blinded to TS result. We compared accuracy of pre-TS and post-TS impression (primary outcome) with McNemar’s test. Test characteristics for treating physician impression were also calculated by dichotomizing acute decompensated heart failure (ADHF), chronic obstructive pulmonary disease (COPD) and pneumonia as present or absent.

**Results:**

57 patients were enrolled with the leading final diagnoses being ADHF (26%), COPD/asthma (30%), and pneumonia (28%). Overall accuracy of the treating physician’s impression increased from 53% before TS to 77% after TS (p=0.003). The post-TS impression was 100% sensitive and 84% specific for ADHF.

**Conclusion:**

In this small study, POCUS evaluation of the heart, lungs and IVC improved the treating physician’s immediate overall diagnostic accuracy for ADHF, COPD/asthma and pneumonia and was particularly useful to immediately exclude ADHF as the cause of acute dyspnea.

## INTRODUCTION

Rapid and accurate diagnosis of the acutely dyspneic patient in the emergency department (ED) is both essential and challenging. Two of the most common causes, acute decompensated heart failure (ADHF) and chronic obstructive pulmonary disease (COPD), differ greatly in both their pathophysiology and treatment, but are often difficult to distinguish clinically in the ED setting.^1^–^5^ Numerous studies indicate that the physical exam, even with the addition of chest radiography, is often inaccurate in differentiating ADHF from COPD/asthma.^1^,^3^–^6^ Moreover, results of advanced diagnostic imaging (computed tomography, consultative echocardiography) and blood tests (particularly brain naturietic peptide [BNP]) are not available during the critical first minutes. Thus, the emergency physician (EP) is often forced to initiate treatment before the etiology of the patient’s respiratory distress can be clearly defined.

Point-of-care ultrasound (POCUS) is emerging as a powerful tool for rapid diagnostic evaluation of ED patients presenting with undifferentiated dyspnea. ADHF, COPD/asthma and other common causes of acute dyspnea all show characteristic findings on POCUS examination of the heart, lungs and inferior vena cava (IVC).^7^–^11^ However, prior studies looking at the examination of each organ individually have generally reported a low specificity in differentiating ADHF from other causes of acute dyspnea.^2^,^12^–^14^ There are limited data on a combined POCUS examination of the heart, lungs and IVC.^15^,^16^ We have developed and refined a rapid multi-organ exam, dubbed “triple scan” (TS), composed of abbreviated echocardiography, lung ultrasound (US) and IVC exam, which can easily be performed by EPs at the bedside of the acutely dyspneic patient.

If the addition of the TS to the history and physical improves the accuracy of the EP’s initial diagnostic impression, its routine use could greatly improve the emergency management of acutely dyspneic patients. The goal of our study was to compare the accuracy of the treating EP’s diagnostic impression before and after results of the TS were available, as compared to final diagnosis.

## METHODS

This was a prospective cohort study involving a convenience sample of ED patients with acute dyspnea. Patients were enrolled from December 2011 through September 2012 in the ED at Alameda Health System – Highland Hospital, an urban academic hospital with approximately 90,000 patient visits per year. During the study period there were a total of 466 patients seen in our ED who were coded as Emergency Severity Index (ESI) level 1–3 acuity presenting with the triage complaint of shortness of breath, asthma, COPD, or congestive heart failure (CHF). Of these patients, it is unknown how many met our specific vital sign inclusion criteria for enrollment.

Criteria for enrollment included age >17 years, a chief complaint of shortness of breath, need for immediate medical intervention to prevent clinical deterioration as judged by the treating physician, and signs of acute respiratory distress at triage, including at least one of the following: respiratory rate >20 breaths per minute; heart rate >100 beats per minute; pulse oximetry <94% on room air. Patients were excluded if the cause of the respiratory distress was associated with trauma or they were able to clearly tell the treating physician what was causing their dyspnea (recurrent asthma, known heart failure, etc.). A post-hoc analysis confirmed that all subjects were ESI level 1 or 2. Patients were enrolled prospectively when any of three EP investigators were available in the ED. These investigator sonologists, two US fellowship-trained attendings and one US fellow, either performed or directly supervised all TS exams. Treating physicians who provided the diagnostic impression could be senior (third- or fourth-year) emergency medicine (EM) residents or attending physicians. Study investigators could enroll patients while on attending shifts; however, in these cases the diagnostic impressions were decided by the treating resident (not the study investigator).

The study protocol was approved by the hospital’s institutional review board. Written consent was obtained from all patients or their healthcare surrogates. Since US evaluations were already considered standard during resuscitations of acutely dyspneic patients, consent for enrollment was obtained after US evaluation and medical stabilization. The TS was performed and images recorded during or immediately after the initial history and physical exam. We used phased array (5-1MHz) and curvilinear (5-2MHz) transducers, at the discretion of the sonologist (SonoSite, Bothell, WA; Micromaxx™, M-Turbo™, or S-FAST™). Although the exact duration of the TS was not documented, exams were generally completed in less than two minutes.

### Echocardiography

We obtained a standard parasternal long axis view and additional parasternal short, subxyphoid, and/or apical four-chamber views as needed. We assessed (a) gross left ventricular ejection fraction, categorized as either normal, poor, or hyperdynamic, estimated by visual gestalt; (b) presence or absence of pericardial effusion and, if present, signs of tamponade physiology (primarily right ventricular diastolic collapse); and (c) presence or absence of right ventricular enlargement (estimated right ventricular chamber size equal to or greater than left ventricular chamber size). This abbreviated echocardiography approach has been previously described in detail by others.^10^,^17^

### Lung Ultrasound

- Using only the bilateral anterior lung windows (representing four lung zones),^18^ we assessed whether there was predominantly an A-line or B-line (indicating abnormal pulmonary fluid) pattern, as described by Lichtenstein.^19^
- We also scanned the lateral chest superior to the hemidiaphragms for the presence or absence of pleural effusions, but were not evaluated for the presence of A or B lines.^18^
- We assessed pleural sliding on 2D and M-mode as needed over the anterior lung fields to exclude pneumothorax.

### Inferior Vena Cava

We obtained either a subxyphoid or right lateral view of the IVC approximately 2cm proximal to the hepatic vein confluence and assessed for IVC collapse during inspiration. The IVC was categorized as plethoric (less than 15% collapse), flat (>90% collapse), or normal (15%–90% collapse), using gross visual estimation.

**Figure 1:**
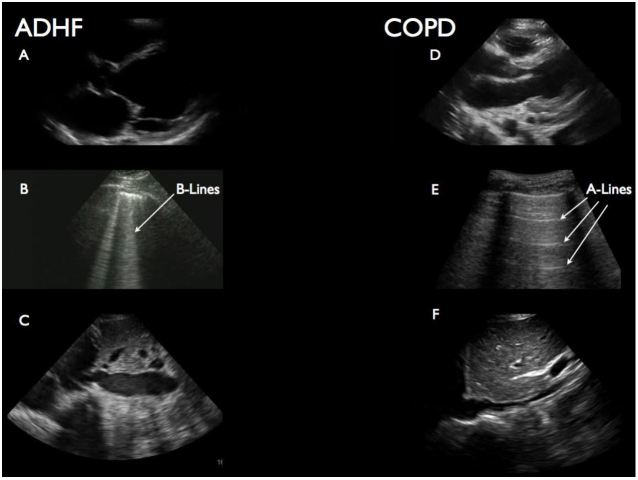
Typical findings on “triple scan” (TS) in acute decompensated heart failure (ADHF) and chronic obstructive pulmonary disease (COPD)/asthma. Images a-c show typical findings of ADHF: dilated left ventricle with poor mitral valve opening (a); vertical b-line artifacts in this case indicating excess lung water (b); dilated inferior vena cava (IVC [lacking respiratory variation]) (c). Images d-e show typical findings in COPD/asthma: normal left ventricle (often hyperdynamic) (d), horizontal a-line artifacts indicating hyperinflation (e) and normal IVC (f).

Study investigators agreed a priori on the ultrasonographic features of the three main diagnoses so that TS results were presented to the treating physician in as standardized a way as possible. ADHF was defined as the presence of B-lines in bilateral anterior lung fields, poor cardiac function, and a non-respirophasic IVC. COPD was suggested by the absence of B-lines in the anterior lung fields with normal or diminished cardiac function and either a non-respirophasic or flat IVC. Examples of sonographic findings in ADHF and COPD are shown in Figure 1. Pneumonia was diagnosed when unilateral B-lines or consolidations were noted on lung US in the setting of hyperdynamic or normal cardiac function and non-plethoric IVC.

After performing a history and physical examination but prior to the TS, and prior to return of other imaging and laboratory results, treating physicians ranked the three most-likely etiologies of the dyspnea (their “pre-TS impression”) and graded their confidence in the leading diagnosis using a Likert scale (1 least confident to 5 most confident). The number one diagnosis was considered the primary impression. We chose, a priori, eight distinct diagnostic categories as the etiology for dyspnea: ADHF, COPD/asthma, pneumonia, acute respiratory distress syndrome (ARDS), pleural effusion, pericardial effusion, pneumothorax, and pulmonary embolism. The treating physicians could also specify a diagnosis other than one of these eight, which was categorized as “other.” Afterwards, the TS was performed by the investigator sonologist. The images were recorded and the sonographic findings were shown to the treating physician, after which the treating physician was again asked to rank their “post-TS impression” and their confidence. The primary outcome measure was the difference in accuracy of the treating physicians’ primary impressions before and after TS. Accuracy was defined as number of cases with correct primary diagnostic impression over total number of cases. We also assessed the test characteristics of treating physician impression for diagnosing ADHF, asthma/COPD and pneumonia before and after TS. We chose these three diseases because they are the most common causes of undifferentiated dyspnea in our ED.

Final diagnosis (criterion standard) was determined by medical record review by two independent senior EM attending physicians with an interest in cardiopulmonary diseases, who had not been involved in patient enrollment. These reviewers were provided with copies of the electronic medical record, including the ED chart, the admitting physician history and physical, all laboratory and radiology reports, consultative echocardiography results, pulmonary function tests, hospital discharge summary and final hospital discharge diagnosis. Only the results of the TS were redacted from the ED chart (blinding to TS result). Reviewer final diagnosis was unstructured and written on a simple data collection sheet. A third chart reviewer was available to adjudicate any disagreement on the final diagnosis, but this was not needed as the reviewers agreed in every case.

We estimated that the treating physician would correctly identify the primary cause of dyspnea 60% of the time. Assuming a power of 0.80, with alpha =0.05, we calculated that we would need to enroll 57 patients to show a 25% improvement in identifying the correct diagnosis after addition of the TS. We report proportions for demographic and clinical variables, means for normally distributed continuous variables (age), and median scores for diagnostic confidence on a five-point Likert scale. McNemar’s test was used to compare correct and incorrect provider impressions pre- and post-TS. We compared pre-TS and post-TS provider impressions as well as diagnostic confidence scores using the Fisher’s exact chi-squared test because of small numbers of observations within some cells. We considered *p*<0.05 to be statistically significant. We calculated test characteristics of provider primary impression by dichotomizing the pre-TS and post-TS impressions and final diagnoses to presence or absence of CHF, then to presence or absence of COPD/asthma and then to presence or absence of pneumonia. We report sensitivity, specificity, positive predictive value, negative predictive value, as well as positive and negative likelihood ratios with 95% CIs for each. Statistical analysis was done using Stata SE version 11 (StataCorp, College Station, TX)

## RESULTS

We enrolled a total of 57 patients with acute dyspnea who met the inclusion criteria. Patient characteristics, clinical course and final diagnoses are presented in Table 1. Twenty-nine of 57 (57%) patients required non-invasive positive pressure ventilation, 48 (84%) were admitted to the hospital and six (10%) to the intensive care unit. Specific ultrasonographic findings among the cohort are listed in Table 2.

**Figure 2:**
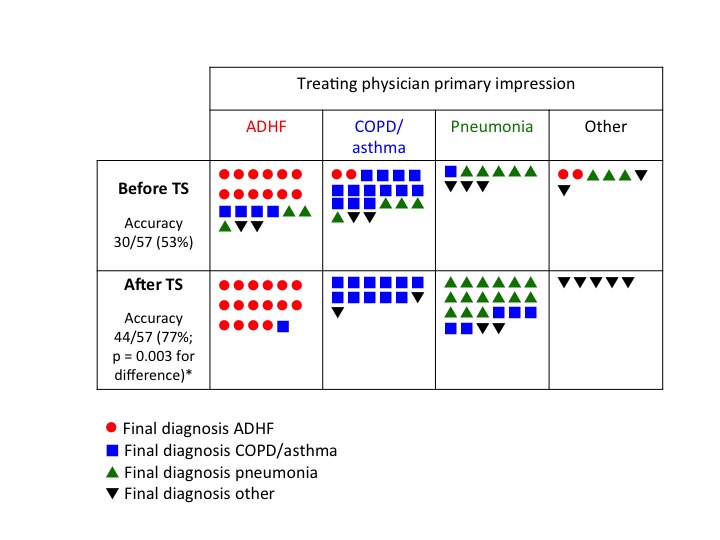
Case level data showing final diagnosis in each case. *ADHF,* acute decompensated heart failure; *COPD,* chronic obstructive pulmonary disease; *TS,* triple scan

Diagnostic accuracy, our primary outcome, improved from 53% before TS to 77% after TS (p=0.003). Case level data, showing the final diagnosis in each case and comparing it to the treating physician’s primary impression (diagnosis rated as most likely), before and after TS, is presented in Figure 2. The treating physician’s primary impression changed after TS in 27 of 57 (47.3%) cases. In 17 of 57 (29.8%) cases, an incorrect impression (pre-TS primary impression not matching the final diagnosis) was changed to the correct diagnosis, whereas in three of 57 (5.2%) of cases the opposite occurred and the treating physician changed a correct impression to an incorrect one. Treating physician’s confidence in their clinical impressions, rated on a five-point Likert scale, improved significantly after the TS (median score 3 before TS versus 5 after TS; p=0.017).

Table 3 compares the test characteristics of the treating physician’s impression, before and after the TS, for diagnosis of ADHF, COPD/asthma and pneumonia. For ADHF, addition of the TS improved the point estimates for both sensitivity (73.3% to 100%) and specificity (78.6% to 95.2%) of the treating physician’s impression. For asthma/COPD the specificity of the impression increased (80.0 to 93.3%), while the sensitivity decreased (76.5% to 64.7%). For pneumonia, sensitivity increased markedly (31% to 100%) while specificity decreased somewhat (90% to 83%). Because of the broad CIs around point estimates, only the improvement in sensitivity for pneumonia reached statistical significance.

## DISCUSSION

In this small cohort study of ED patients presenting with acute dyspnea requiring rapid intervention, an abbreviated multi-organ POCUS examination of the heart, lungs and IVC, which we named the “Triple Scan,” significantly improved physician diagnostic accuracy in determining the correct etiology of dyspnea. In particular we found that, in conjunction with history and physical exam, the TS excluded ADHF with 100% sensitivity – within just a few minutes of presentation. Two distinguishing strengths of our study are that in our cohort there was a high proportion of severe disease requiring noninvasive ventilation and hospital admission, and that our simplified TS protocol can be performed rapidly and the results incorporated into the working clinical impression within minutes of presentation, prior to chest radiograph or blood test results.

Initiating immediate, targeted therapy for ADHF, COPD, pneumonia and other causes of acute dyspnea is important; however, correctly identifying the cause of dyspnea in a clinically unstable patient can be challenging. The physical exam, even with the addition of chest radiography, is often inaccurate,^1^,^6^ and simply starting “dual therapy” for ADHF and COPD can be harmful.^20^,^21^ Results of laboratory studies, such as BNP, and consultative echocardiography are not available in the immediate setting. Furthermore, BNP can be elevated in the setting of CHF when an etiology other than ADHF actually accounts for the acute dyspnea.^22^,^23^ A symptom-based POCUS exam that could be performed by EPs within minutes of presentation, that substantially improved diagnostic accuracy, would be a major step forward in the ED management of acute dyspnea.

Use of abbreviated POC echocardiography to evaluate dyspnea in the ED setting was first introduced by Kimura in 2001,^24^ and soon after, studies were published showing that EPs can reliably and rapidly assess ejection fraction and presence of pericardial effusion.^25^–^27^ Over the following decade studies appeared in the EM literature of POC lung US to assess for extravascular lung water (B lines, or comet tails) and hyperinflation (prominent A lines) and assessment of IVC collapsibility to gage volume status.^28^,^29^ In addition, numerous reports have shown that POCUS can rapidly identify pneumothorax, signs of pulmonary embolus and pneumonia.^30^–^32^

To date, there have been five EM studies evaluating a multi-organ POCUS protocol similar to our TS – combining abbreviated echocardiography, lung US and IVC assessment – in the setting of undifferentiated dyspnea. Three studies focused strictly on diagnosis of ADHF.^2^,^12^,^16^ With regard to the test performance characteristics of POCUS as a stand-alone test for ADHF, Kajimoto et al. found a sensitivity and specificity of 94% and 91%; Anderson et al. found a sensitivity of only 34% and specificity of 91%; and Russell et al. reported sensitivity and specificity of 83% and 83%. Russell et al. also found that the specificity of treating physician diagnosis for ADHF improved from 44% to 83% when POCUS was used. Two studies published in 2014, like ours, assessed the impact of multi-organ POCUS, in addition to history and physical, on the accuracy of the treating physician’s initial diagnosis. In a randomized controlled trial (RCT) where patients were randomly assigned to initial assessment with and without POCUS, Pirozzi et al. found that the rate of discordance between initial and final diagnosis was 5% in the POCUS group compared to 50% in the control group.^15^ Lauresen et al. performed an RCT involving a somewhat different multi-organ POCUS protocol that included proximal DVT assessment instead of IVC assessment, and allowed treating physicians to see other diagnostic test results before giving a diagnostic impression at four hours.^33^ These authors found a proportion of correct presumptive diagnosis in the POCUS group of 88% compared to 63.7% in the control group, a significant difference.

Our study supports the findings of these other recent publications regarding the value of POCUS to correctly diagnose ADHF. Like Russell et al., we found that POCUS is useful in reducing false positive clinical ADHF diagnosis. In other words, while treating physicians tended to initially “overcall” ADHF, a POCUS showing no signs of ADHF forced them to consider other diagnoses, improving their diagnostic specificity for ADHF. Our study also found that POCUS increases treating physician sensitivity for ADHF, enabling them to pick up subtle ADHF cases initially misdiagnosed as COPD or other diagnoses. This is consistent with the 91% sensitivity reported by Kajimoto and with a recent meta-analysis that reported a summary sensitivity of 94% of POCUS for ADHF diagnosis.^2^,^34^

Yet our study went beyond just ADHF diagnosis. Similar to the studies by Pirrozi and Laursen, we found that COPD/asthma and pneumonia were roughly as common as ADHF in our acutely dyspneic patients, and that POCUS generally improved the treating physician’s ability to make these diagnoses too.^15^,^33^ With regard to COPD/asthma, our treating physicians initially “overcalled” this diagnosis in eight cases, in which POCUS revealed unexpected ADHF in two and findings correctly indicating pneumonia in four. This underscores the notion that “all that wheezes is not asthma” and shows that there is a subgroup of patients with wheezing on exam (usually from COPD) who have concomitant pneumonia, which may be the true cause of their acute dyspnea. The improvement in COPD/asthma specificity, however, came at a cost of somewhat reduced sensitivity; we discovered that the finding of focal B-lines in patients with COPD, while sometimes a subtle sign of pneumonia, also lead to a false positive impression of pneumonia in four cases. Because of small sample size, neither the change in sensitivity or specificity reached statistical significance.

The improvement in our treating physicians’ ability to diagnose pneumonia following TS is similar to the findings of Pirrozi et al.^15^ This likely reflects both direct diagnosis, when sonographic findings indicating pneumonia were seen, such as focal B-lines, as well as indirect diagnosis, when absence of ADHF findings forced consideration of an alternative diagnosis. The high diagnostic accuracy for pneumonia (sensitivity 100%, specificity 83%) as compared to the hospital discharge diagnosis gold standard (which takes into account chest radiograph and often computed tomography [CT] findings) is not surprising; POCUS has been found to be highly accurate as a stand-alone test for pneumonia in children and adults and outperforms chest radiograph when compared to a CT gold standard.^31^,^35^,^36^ Our findings suggest that POCUS can point clinicians toward a correct diagnosis of pneumonia early in the evaluation of the dyspneic patient, when it might otherwise be missed because the patient is initially afebrile, or wheezing, or assumed to have ADHF, or because the portable chest radiograph lacks an obvious infiltrate.

It is important to note that we accomplished these improvements in diagnostic accuracy using a highly abbreviated POCUS exam that was usually performed within minutes of arrival on patients who were frequently in extremis. As opposed to the more comprehensive and time-consuming echocardiography protocols used by other investigators,^12^,^16^,^33^ the echocardiography component of our TS protocol simply focused on ejection fraction by gross visual estimation (an accepted method), presence or absence of pericardial effusion and right ventricular enlargement.^10^ A single view was often adequate to assess these questions. Similarly, the lung exam consisted of assessment of only three anterior lung fields bilaterally rather than the eight zones specified in most other protocols.^12^,^15^,^33^ Not only is such an abbreviated protocol feasible during initial resuscitation of the sickest dyspneic patients, but it is likely to be more generalizable to non-expert sonographers.

## LIMITATIONS

Our study has numerous limitations. Convenience sampling and the small number of subjects limit the strength of our conclusions. Although we believe our abbreviated TS protocol can be performed rapidly and that scan results are reproducible, we did not measure the time required to perform the TS or measure intra-observer reliability for performing and interpreting TS. This was a pragmatic study of the impact of real-time TS on clinician diagnostic accuracy and we were not assessing accuracy of the TS result itself. The scans were performed by investigators who were attending physicians with an interest in cardiopulmonary diseases, who may have acted as bedside consultants to the treating physicians between formation of their pre-TS and post-TS impressions. Treating physicians were not blinded to the study objective. The criterion standard of final diagnosis based on chart review, though typical and well accepted in studies such as this, is always problematic.^33^ Our analysis focused on the primary diagnosis (listed by the treating physician and the blinded chart reviewers as the number one/most likely cause of dyspnea), but in many cases multiple diagnoses were listed, which reflects the reality that many subjects presented with multiple disease processes, such as ADHF plus COPD or COPD plus pneumonia. Our analysis does not account well for this overlap of multiple diagnoses.

A major limitation of this study, like all studies to date of POCUS for dyspnea, is that USs were performed and interpreted by expert sonographers. This limits external validity, particularly to non-academic ED settings. The study we would like to see is a pragmatic trial involving non-expert sonographer community EPs and trainees, in which patients are randomized to initial evaluation with POCUS versus without POCUS, with USs performed by the actual treating physician, and with outcomes such as use of other diagnostic tests and ED throughput, as well as physician diagnostic accuracy.

## CONCLUSION

In a cohort of patients with severe, undifferentiated dyspnea, immediate TS – in essence, an extension of the physical exam – resulted in a statistically significant improvement in treating physicians’ overall diagnostic accuracy. While its primary utility appeared to be rapid diagnosis or exclusion of ADHF, the TS also seemed to markedly improve the diagnosis of pneumonia, though these findings did not reach statistical significance. Taken together with the results of other recent studies, it seems fair to conclude that multi-organ POCUS should become a routine part of the ED evaluation of acute dyspnea.

## Figures and Tables

**Table 1:** Patient characteristics, clinical course and final diagnoses in a study evaluating utility of point-of-care ultrasound. Total N=57.

	n	%
Characteristics		
Age, mean years	58.2	
Male	36	63
Noninvasive ventilation	29	57
Admitted to hospital	48	84
Admitted to ICU	6	10
Died during admission	1	1.8
Final diagnosis		
ADHF	15	26.3
Asthma/COPD	17	29.8
Pneumonia	16	28.1
Obstructive sleep apnea	3	5.2
Pulmonary embolus	2	3.5
ARDS	1	1.8
Pleural effusion	1	1.8
Interstitial lung disease	1	1.8
Psychogenic	1	1.8

*ICU,* intensive care unit; *ADHF,* acute decompensated heart failure; *COPD,* chronic obstructive pulmonary disease; *ARDS,* acute respiratory distress syndrome

**Table 2:** Ultrasonographic findings among all 57 patients.

	n	%
Ultrasonographic finding		
Hyperdynamic LV function	15	26.3
Decreased LV systolic function	18	31.6
Pericardial effusion	2	3.5
Cardiac tamponade	--	--
A-lines bilateral	22	38.5
B-lines bilateral	22	38.5
B-lines unilateral	13	22.9
Pleural effusion	3	5.3
Lack of pleural sliding	--	--
Plethoric IVC	20	35.1
Flat IVC	19	33.3

*LV,* left ventricular; *IVC,* inferior vena cava

**Table 3:** Test characteristics for treating physician primary impression, before and after “triple scan” (TS).

	Before TS		After TS	
	
	Value	95% CI	Value	95% CI
CHF				
Sensitivity, %	73.3	44.9–92.2	100.0	78.2–100.0
Specificity, %	78.6	63.2–89.7	95.2	83.8–99.4
LR positive	3.4	1.8–6.6	21.0	5.4–81.2
LR negative	0.3	0.1–0.8	0.0	0.0–0.5
COPD/Asthma				
Sensitivity, %	76.5	50.1–93.2	64.7	38.3–85.8
Specificity, %	80.0	64.4–90.9	93.3	77.9–99.2
LR positive	3.8	2.0–7.5	9.7	2.4–38.7
LR negative	0.3	0.1–0.7	0.4	0.2–0.7
Pneumonia				
Sensitivity, %	31.2	11.0–58.7	100.0	78.2–100
Specificity, %	90.2	76.9–97.3	82.9	67.9–92.8
LR positive	3.2	0.98–10.44	5.9	3.0–11.5
LR negative	0.76	0.54–1.08	0.0	0.0–0.5

*CHF,* congestive heart failure; *COPD,* chronic obstructive pulmonary disease; *LR,* likelihood ratio

## References

[1] Collins SP, Lindsell CJ, Peacock WF (2006). Clinical characteristics of emergency department heart failure patients initially diagnosed as non-heart failure. BMC Emerg Med.

[2] Kajimoto K, Madeen K, Nakayama T (2012). Rapid evaluation by lung-cardiac-inferior vena cava (LCI) integrated ultrasound for differentiating heart failure from pulmonary disease as the cause of acute dyspnea in the emergency setting. Cardiovasc Ultrasound.

[3] Knudsen CW, Omland T, Clopton P (2004). Diagnostic value of B-Type natriuretic peptide and chest radiographic findings in patients with acute dyspnea. Am J Med.

[4] McCullough PA, Nowak RM, McCord J (2002). B-type natriuretic peptide and clinical judgment in emergency diagnosis of heart failure: analysis from Breathing Not Properly (BNP) Multinational Study. Circulation.

[5] Wang CS, FitzGerald JM, Schulzer M (2005). Does this dyspneic patient in the emergency department have congestive heart failure?. JAMA.

[6] Jang TB, Aubin C, Naunheim R (2012). The predictive value of physical examination findings in patients with suspected acute heart failure syndrome. Intern Emergency Med.

[7] Blehar DJ, Dickman E, Gaspari R (2009). Identification of congestive heart failure via respiratory variation of inferior vena cava diameter. The American journal of emergency medicine.

[8] Cibinel GA, Casoli G, Elia F (2012). Diagnostic accuracy and reproducibility of pleural and lung ultrasound in discriminating cardiogenic causes of acute dyspnea in the emergency department. Intern Emergency Medicine.

[9] Gheorghiade M, Follath F, Ponikowski P (2010). Assessing and grading congestion in acute heart failure: a scientific statement from the acute heart failure committee of the heart failure association of the European Society of Cardiology and endorsed by the European Society of Intensive Care Medicine. Eur J Heart Fail.

[10] Labovitz AJ, Noble VE, Bierig M (2010). Focused cardiac ultrasound in the emergent setting: a consensus statement of the American Society of Echocardiography and American College of Emergency Physicians. J Am Soc Echocardiogr.

[11] Lichtenstein D, Meziere G (1998). A lung ultrasound sign allowing bedside distinction between pulmonary edema and COPD: the comet-tail artifact. Intensive Care Med.

[12] Anderson KL, Jenq KY, Fields JM (2013). Diagnosing heart failure among acutely dyspneic patients with cardiac, inferior vena cava, and lung ultrasonography. Am J Emerg Med.

[13] Gargani L (2011). Lung ultrasound: a new tool for the cardiologist. Cardiovasc Ultrasound.

[14] Mantuani D, Nagdev A, Stone M (2012). Three-view bedside ultrasound for the differentiation of acute respiratory distress syndrome from cardiogenic pulmonary edema. Am J Emerg Med.

[15] Pirozzi C, Numis FG, Pagano A (2014). Immediate versus delayed integrated point-of-care-ultrasonography to manage acute dyspnea in the emergency department. Crit Ultrasound J.

[16] Russell FM, Ehrman RR, Cosby K (2015). Diagnosing acute heart failure in patients with undifferentiated dyspnea: a lung and cardiac ultrasound (LuCUS) protocol. Acad Emerg Med.

[17] Weekes AJ, Quirke DP (2011). Emergency echocardiography. Emerg Med Clin North Am.

[18] Volpicelli G, Caramello V, Cardinale L (2008). Diagnosis of radio-occult pulmonary conditions by real-time chest ultrasonography in patients with pleuritic pain. Ultrasound Med Biol.

[19] Lichtenstein D, Meziere G, Biderman P (1997). The comet-tail artifact. An ultrasound sign of alveolar-interstitial syndrome. Am J Respir Crit Care Med.

[20] Dharmarajan K, Strait KM, Lagu T (2013). Acute decompensated heart failure is routinely treated as a cardiopulmonary syndrome. PloS one.

[21] Singer AJ, Emerman C, Char DM (2008). Bronchodilator therapy in acute decompensated heart failure patients without a history of chronic obstructive pulmonary disease. Ann Emerg Med.

[22] Komiya K, Ishii H, Murakami J (2012). Relationship between CT findings and the plasma levels of brain natriuretic peptide in 29 patients with acute cardiogenic pulmonary edema. Acad Radiol.

[23] Nakane T, Kawai M, Komukai K (2012). Contribution of extracardiac factors to the inconsistency between plasma B-type natriuretic peptide levels and the severity of pulmonary congestion on chest X-rays in the diagnosis of heart failure. Intern Med.

[24] Kimura BJ, Bocchicchio M, Willis CL (2001). Screening cardiac ultrasonographic examination in patients with suspected cardiac disease in the emergency department. Am Heart J.

[25] Mandavia DP, Hoffner RJ, Mahaney K (2001). Bedside echocardiography by emergency physicians. Ann Emerg Med.

[26] Moore CL, Rose GA, Tayal VS (2002). Determination of left ventricular function by emergency physician echocardiography of hypotensive patients. Acad Emerg Med.

[27] Randazzo MR, Snoey ER, Levitt MA (2003). Accuracy of emergency physician assessment of left ventricular ejection fraction and central venous pressure using echocardiography. Acad Emerg Med.

[28] Nagdev AD, Merchant RC, Tirado-Gonzalez A (2010). Emergency department bedside ultrasonographic measurement of the caval index for noninvasive determination of low central venous pressure. Ann Emerg Med.

[29] Volpicelli G, Mussa A, Garofalo G (2006). Bedside lung ultrasound in the assessment of alveolar-interstitial syndrome. Am J Emerg Med.

[30] Dresden S, Mitchell P, Rahimi L (2014). Right ventricular dilatation on bedside echocardiography performed by emergency physicians aids in the diagnosis of pulmonary embolism. Ann Emerg Med.

[31] Shah VP, Tunik MG, Tsung JW (2013). Prospective evaluation of point-of-care ultrasonography for the diagnosis of pneumonia in children and young adults. JAMA Pediatr.

[32] Wilkerson RG, Stone MB (2010). Sensitivity of bedside ultrasound and supine anteroposterior chest radiographs for the identification of pneumothorax after blunt trauma. Acad Emerg Med.

[33] Laursen CB, Sloth E, Lassen AT (2014). Point-of-care ultrasonography in patients admitted with respiratory symptoms: a single-blind, randomised controlled trial. Lancet Respir Med.

[34] Al Deeb M, Barbic S, Featherstone R (2014). Point-of-care ultrasonography for the diagnosis of acute cardiogenic pulmonary edema in patients presenting with acute dyspnea: a systematic review and meta-analysis. Acad Emerg Med.

[35] Liu XL, Lian R, Tao YK (2015). Lung ultrasonography: an effective way to diagnose community-acquired pneumonia. EMJ.

[36] Ye X, Xiao H, Chen B (2015). Accuracy of Lung Ultrasonography versus Chest Radiography for the Diagnosis of Adult Community-Acquired Pneumonia: Review of the Literature and Meta-Analysis. PloS one.

